# Investigating the use of patient involvement and patient experience in quality improvement in Norway: rhetoric or reality?

**DOI:** 10.1186/1472-6963-13-206

**Published:** 2013-06-06

**Authors:** Siri Wiig, Marianne Storm, Karina Aase, Martha Therese Gjestsen, Marit Solheim, Stig Harthug, Glenn Robert, Naomi Fulop

**Affiliations:** 1 Department of Health Studies, University of Stavanger, N-4036, Stavanger, Norway; 2 Faculty of Health Studies, Sogn og Fjordane University College, Førde, Norway; 3 Department of Research and Development, Førde Central Hospital, Førde, Norway; 4 Department of Research and Development, Haukeland University Hosptial, NO- 5021, Bergen, Norway; 5 Insitute of Medicine, University of Bergen, NO- 5021, Bergen, Norway; 6 National Nursing Research Unit, King’s College London, 57 Waterloo Road, London SE1 8WA, UK; 7 Department of Applied Health Research, University College London, 1-19 Torrington Place, London WC1E 7HB, UK

**Keywords:** Patient experience, Patient involvement, Quality improvement, Multi-level study

## Abstract

**Background:**

Patient involvement in health care decision making is part of a wider trend towards a more bottom-up approach to service planning and provision, and patient experience is increasingly conceptualized as a core dimension of health care quality.

The aim of this multi-level study is two-fold: 1) to describe and analyze how governmental organizations expect acute hospitals to incorporate patient involvement and patient experiences into their quality improvement (QI) efforts and 2) to analyze how patient involvement and patient experiences are used by hospitals to try to improve the quality of care they provide.

**Methods:**

This multi-level case study combines analysis of national policy documents and regulations at the macro level with semi-structured interviews and non-participant observation of key meetings and shadowing of staff at the meso and micro levels in two purposively sampled Norwegian hospitals. Fieldwork at the meso and micro levels was undertaken over a 12-month period (2011–2012).

**Results:**

Governmental documents and regulations at the macro level demonstrated wide-ranging expectations for the integration of patient involvement and patient experiences in QI work in hospitals. The expectations span from systematic collection of patients’ and family members’ experiences for the purpose of improving service quality through establishing patient-oriented arenas for ongoing collaboration with staff to the support of individual involvement in decision making. However, the extent of involvement of patients and application of patient experiences in QI work was limited at both hospitals. Even though patient involvement was gaining prominence at the meso level − and to a lesser extent at the micro level − relevant tools for measuring and using patient experiences in QI work were lacking, and available measures of patient experience were not being used meaningfully or systematically.

**Conclusions:**

The relative lack of expertise in Norwegian hospitals of adapting and implementing tools and methods for improving patient involvement and patient experiences at the meso and micro levels mark a need for health care policymakers and hospital leaders to learn from experiences of other industries and countries that have successfully integrated user experiences into QI work. Hospital managers need to design and implement wider strategies to help their staff members recognize and value the contribution that patient involvement and patient experiences can make to the improvement of healthcare quality.

## Background

The Norwegian healthcare system is undergoing significant reform, including an increasing focus on quality, of which safety is an important component. Part of the government’s focus, as in many countries, is related to incorporating patient experiences and strengthening the patients’ role in the improvement of care quality [1-3]. As part of its increased attention to quality and safety, Norway launched its first national patient safety campaign in 2011, and the first report to the Parliament on quality and safety in healthcare was issued in 2012.

In the literature, concepts such as patient centeredness, shared decision making, and patient experiences are presented as vital dimensions of improving healthcare quality in practice and for empowering patients [4-7]. Each represents an important ideological counterpart challenging paternalism and disease-oriented models of care as they relate to the person, their experiences and broader health status. Patient involvement and patient experience in QI is part of a wider trend towards a more bottom-up approach of service planning and provision [5]. Governmental strategies, rules, and regulations at the macro (policy) level raise expectations that services at the meso (organizational) and micro (clinical services) levels will be commissioned based on their ability to provide patient-centered care and offer patients a choice of provider. Such ambitions call for greater focus on how organizations and practitioners organize the collection and use of patient experience data in QI initiatives to fulfill these expectations [8]. Methods and techniques such as patient surveys and checklists can be applied to integrate patient experiences and involve patients in QI. Theoretically, patient surveys and other methods seem to be useful sources of feedback for services, but research shows that their use in QI is not straightforward [8,9]. Previous research has shown that QI based on patient experience has not been made a priority in many hospitals, and only a few of these have adequate systems for coordinating the collection of such data, assessing its importance and implications and acting on the results in a systematic way [10]. A study of QI strategies in Europe revealed that monitoring patients’ views by systematically conducting patient surveys was common practice in 64.5% of the 389 participating European hospitals but with widely disparate practice in different countries [11]. Although local and national patient survey data are available in some countries, few staff members use such data to guide service improvement, and some skepticism is expressed about their validity for local QI efforts [12].

### Aim

Further research is needed about the interactions between policy and practice related to how patient involvement and patient experiences are incorporated into QI in diverse national and cultural contexts. This study seeks to increase knowledge about the macro-, meso-, and micro-level relationships concerning patient involvement and the use of patient experiences in QI in Norway. The overall aim is two-fold: 1) to describe and analyze how Norwegian governmental organizations expect acute hospitals to incorporate patient involvement and patient experiences into their QI efforts, and 2) to analyze how patient involvement and patient experiences are used to improve quality in practice by Norwegian hospitals.

This paper reports on Norwegian macro-level expectation and regulations in place to ensure the integration of patient involvement and patient experiences in improving quality within acute hospitals. The paper then reports on how these macro-level expectations are translated into practice in two purposively selected Norwegian public hospitals and discusses the challenges and obstacles facing managers and employees at both the meso and micro levels of those organizations.

### Concepts

As Bate & Robert [13] suggest, involving patients in QI and listening and responding to what they say has become an accepted part of attempts to improve hospital services over the past five years. Consequently, terms such as ‘patient and public involvement’ (PPI), ‘user involvement’, ‘patient-centered care’, and ‘co-design’ are commonplace in health policy circles. In addition to these various concepts, various methods of involving patients and service users in QI in health care are available. Patients have attended stakeholder events, participated in discovery interviews, completed surveys, mapped healthcare processes, and even designed new hospitals with healthcare staff. However, to date, efforts have not necessarily focused on the patients’ experience beyond asking what was good and what was not [13]. Patient-centered care, shared decision-making, and patient participation are different conceptualizations of this movement, but all seek to incorporate patients’ experiences and perspectives on their treatment and care in efforts to improve quality. Although these conceptualizations are advocated in the literature, they have been subject to empirical research to varying degrees [14,15].

*Patient-centered care* − in addition to representing a means towards health care quality − is desirable in its own right [15,16]. The term ‘patient-centered’ has been used to describe an approach in which the therapist ‘sees the situation through the eyes of the client’, attends to patients’ experiences with their illness, empathizes with their feelings and fears [15], or refers to professionals creating opportunities for and responding to patients’ desires for information and participation in treatment decision making [17]. Although patient-centeredness is advocated and integrated in the training of health care providers, there has been a relatively poor understanding of how to promote and measure its core components [15]. The most examined interventions have focused on enhancing the provider-patient communication in clinical consultations. Lewin et al. [15] conclude in their review that training of providers in patient-centeredness may improve communication with patients, enable clarification of patients’ concerns, and improve satisfaction with care. However, it remains unclear if training makes a difference to health care use or outcomes for patients and how this affects quality of health care [15].

*Shared decision making* promotes the right for patient involvement in decisions concerning own health and has been cited as one important tool in order to redesign services to improve quality of health care [4]. Shared decision making implies that the patients bring their experiences and opinions to the clinical encounter, and healthcare professionals provide information, lay out options, present their potential consequences, and explore the patient’s experiences, expectations, and worries about treatment and care [18]. It has been argued that the model has not been widely implemented in the clinical field. Reported barriers to implementation are time constraints and inapplicability of intervention due to the patients’ characteristics or clinical situation. Facilitators to implementation are provider motivation and interventions’ positive impact on clinical process or patient outcome [14].

*Patient participation* pertains to the patients’ involvement and role in decision making in matters relating to their own treatment and care; the term is often used in relation to concepts such as patient involvement, partnership, and patient control [18]. A common way of conceptualizing participation has been to refer to different categories of patient participation combined with involvement of the individual in health care decision making [19]. Patient participation also addresses involvement at the service level, including patients in programs and QI initiatives such as service user panels or advisory boards, or involving patients in provider training. A key issue is to ensure that people’s experiences and perspectives on service provision are heard and preferably taken into account, for example, in decision making about service development [20].

*Co-design* draws its inspiration from a subfield of the design sciences (which include architecture and software engineering) whose distinctive features are (a) direct patient and provider participation in a face-to-face collaborative venture to co-design services, and (b) a focus on designing experiences as opposed to systems or processes [21]. Ethnographic methods such as observation and narrative interviews are thus preferred. Co-design makes two particular contributions to QI thinking. Firstly, it offers a new lens, or frame of mind, through which to conceive approaches to improving patient experiences of healthcare; primarily its pragmatic nature highlights the importance of making sense of the experience and finding solutions to poorly designed interactions. Secondly, it offers methods, tools. and techniques (such as modeling and prototyping) which were little used in health care improvement work until very recently [13]*.*

Tritter [17] has developed a framework for organizing these concepts and illustrates how they operate at different levels of health care. He uses the following three dualities: a) direct and indirect involvement, b) patient involvement operating at the individual and collective level, and c) patient involvement being reactive or proactive. Direct involvement at the individual level refers to a patient’s participation, emphasizing the patient’s views and experiences in shared decision making about his or her treatment. Direct collective involvement may include patient support groups for designing services (a key feature of co-design). People’s views are sought and included in actual decision-making tasks. Indirect involvement refers to activities generating information from patients through, for example, patient experience surveys, but during which the decision on how to act on this information still remains in the hands of health professionals. In terms of the reactive or proactive nature of involvement, the former refers to involvement in response to a pre-defined agenda whilst the latter implies a greater role in shaping the agenda [17].

Gathering patient experiences of healthcare services is one type of patient involvement activity; others include, for example, self-management or self-monitoring of one’s own health. There has been a relatively recent proliferation in the methods and approaches for capturing patient experience that have been implemented by individual hospitals to measure patient experiences [10,22]. These include ward-level surveys, interviews and focus groups, patient forums, informal feedback to patient advocacy groups, formal complaints, comments on websites, and feedback on the performance of individual clinicians for appraisal or revalidation purposes. The value of these initiatives lies in an increased sense of local ownership and service relevance. Numerous studies have reported improvements following systematic gathering of patient feedback by hospitals [9,13,23]. However, the implementation of local methods and approaches often lacks standard definitions of questions, thus creating difficulties in making sense of a plethora of data points that cannot be used to compare with other services/organizations or within the service over time [24].

## Methods

This study is part of the EU FP7 project “Quality and Safety in European Union Hospitals: A Research-Based Guide for Implementing Best Practice and a Framework for Assessing Performance (QUASER)” (for details of the study protocol and hospital selection process, see [25,26]).

### Design and sample

QUASER is an in-depth multi-level analysis of healthcare quality policies and practices including longitudinal case studies in a sample of 10 hospitals in five countries (Norway, England, Portugal, Sweden, and the Netherlands). Two central features of QUASER are 1) the definition of quality, incorporating three aspects: patient experience, patient safety, and clinical effectiveness and 2) to study quality from a multi-level perspective incorporating the macro (national healthcare system), meso (hospital), and micro (frontline clinical team) levels [25,27]. This article covers patient experiences and patient involvement in QI in the Norwegian case study. Two Norwegian hospitals were selected and approached in 2010 based on their performance regarding several national quality indicators. One of the hospitals was determined to be ‘high performing’ while the other hospital was deemed ‘developing’. Both case study hospitals (referred to as Site A and Site B) are studied at the meso and micro levels. The study includes two clinical micro systems at the high performing Site A (maternity and oncology) and one clinical micro system at the developing Site B (maternity).

The research has been performed with the ethical approval of the Norwegian Social Science Data Services (May 5, 2011, Ref. 26636).

### Data collection

The data collection at the macro level includes acquisition of national policy documents (e.g. regulation, national health plan, national QI strategy, and Whitepapers) developed by macro-level organizations in the Norwegian healthcare system (e.g. Ministry of Health and Care Services, Directorate of Health, and Norwegian Board of Health Supervision). We analyzed three policy documents (two national health plans [year 2007–2010 and year 2011–2015] and the current national QI strategy) and six legal acts. A legal act on mental health care was relevant but excluded, as this study covers acute health care. All documents are publicly available on the Internet. Our data collection at the meso and micro levels involves the triangulation of methods and researchers. The study includes semi-structured interviews (97), focus group interviews (2), an interview with the Health and Care Ombudsman (in the region of Site B), shadowing of staff and observation of key meetings and QI projects (45 hours), and analysis of key documents. Examples of observed meetings and QI projects include shift handover and morning meetings at the micro level, patient safety committee meetings, management meetings, quality conferences at the meso level and QI projects related to safe surgery, and to transfer of patients from specialized to primary healthcare services. Table 1 summarizes the details about the data collection methods, number of informants, and hours of observation at each level within the case hospitals. All interviews were audiotaped and transcribed.

**Table 1:** Data collection activities and data sources

**System level**	**Site A**	**Site B**
Meso level	**Interviews**: 18	**Interviews**: 25
	(17 senior managers and one user representative).	(23 senior managers, one user representative, one patient ombudsman^1^)
	**Observations**: 2 hours	**Observations**: 2 hours
Micro level	**Interviews**: 25	**Interviews**: 16
	(Clinical staff and middle managers - Maternity: 14 informants and oncology: 11 informants)	(Clinical staff and middle managers - Maternity)
	**Focus group interview**: 1 (3 informants, maternity).	**Focus group interview**: 1 (7 informants, maternity)
	**Observation**: 20 hours (Maternity: 10 hours, Oncology: 10 hours).	**Observation**: 12 hours (Maternity)
QI project	**Interviews**: 7	**Interviews**: 6
	**Observation**: 2 hours	**Observation**:7 hours

^1^Patient ombudsman is a public body and is not part of the hospital organization.

### Analysis

At the macro level, we analyzed regulations and policy documents to map the stated governmental expectations and to explore the use of different concepts related to patient experiences and patient involvement in improving the quality of care. The role of the macro-level data is to link the governmental expectations of how these are addressed by the hospitals at meso and micro level. The interview guides at the meso and micro levels covered six topics (structure, politic, culture, emotions, education, and physical environment and technology) based on Bate et al. [28]. In the interview guides, the six topics were linked to the three aspects of quality (clinical effectiveness, patient safety, and patient experiences) defined in the QUASER study. This paper focuses on the patient experience component and how patients are involved in QI. We analyzed how the meso and micro level relate to macro-level expectations regarding the incorporation of patient involvement and patient experience in QI according to Tritter’s framework in terms of a) direct or indirect approaches, b) actions at the individual or collective (system) level, and 3) whether or not involvement is approached reactively or proactively [17]. The multilevel design and analysis are conducted to understand the role of interaction between levels in QI. To ensure trustworthiness in the analysis, we have applied analyst triangulation and member checks [29,30]. In our analytical process, the researchers discussed and refined the analysis according to Tritter’s framework [17] and emerging themes in the data [31]. All interview transcripts and field notes from the meso and micro level were uploaded and analyzed by using Nvivo.

### Limitations

The QUASER project seeks to understand how hospitals organize for involving patients and use patient experiences in QI; hence patients are not directly included as sources in the data collection and in the ethical approval [25]. The voice of the patients is ensured by including user representatives in the data collection at both hospitals and representatives from the Health and Care Ombudsman. Furthermore, the data material does not involve real-time observation of user panel meetings, but minutes from these meetings and interviews with the user panel representatives are included. Lastly, Norway launched a report to the Parliament on quality and safety in healthcare in December 2012. This report is not included in the analysis due to the fact that data collection at the hospitals’ meso and micro levels was accomplished in April 2012. Further data collection will be needed in order to realize the effects of the report to the Parliament on the two hospitals.

## Results

Our results firstly describe the governmental expectations, as these are presented in regulations and vital policy documents in the Norwegian healthcare system. Then we provide a short contextual description of the two hospitals before presenting the results related to patient involvement and the use of patient experiences in the QI practices of the two case study hospitals.

### Governmental expectations

Our analysis of the three Norwegian macro-level policy documents and the six legal acts demonstrates a clear commitment to the comprehensive rights and obligations related to patient involvement. Patient involvement is regulated in a two-sided approach, stating patients’ rights on the one hand and service providers’ obligations on the other. The Patient Right Act (1999) regulates patient involvement and information exchange; patients have the right to be directly involved in decisions regarding their own treatment at the individual level, and patient involvement should be adjusted to the patients’ capability of providing and receiving information [32]. The Specialized Health Care Act (1999), the Health Trust Act (2001), and the Health and Care Service Act (2011) regulate the specialist and primary healthcare services,^a^ obligation to include patients in planning and decision making regarding their individual treatment and to ensure the systematic collection and use of patient experiences at an organizational level [33-35]. The regulation of quality and safety in Norwegian healthcare is based on enforced self-regulation [36-38]. The healthcare law and regulation link the use of patient experience to QI. According to the Supervisory Act (1984) and requirement in the Internal Control Regulation for Health and Social Services (2002; §4e), the enforced self-regulation approach in Norway requires the use of patient experiences and experiences from the next of kin in improving quality of care [39,40], but the regulation does not specify how to approach and incorporate these issues in the systematic QI work.

National policy documents demonstrate wide-ranging governmental expectation at the macro level for a) using patient experiences in QI, b) the future role of patients, and c) patient involvement in decision making at all levels [2,3,41]. The National Healthcare Plans (NHCP) (2007–2010 and 2011–2015) [2,41] underline the new role of patients as experts on their own health and the need for a stronger emphasis on patient involvement [41]:p.245. Objectives of the current NHCP (2011–2015) aim at more proactive involvement and collaboration with patients and patient organization in service development, action plans, and development of national guidelines [2]: p.85. Expectations are also stated in a direction where patient experiences should be beneficial for other service users [41]:p.245, and patient involvement should be incorporated at all levels of the healthcare system − at the policy level, system level, and individual level [2]:p.86, [41]:p.273.

The link between QI and use of patient experiences are obvious in both plans for the period from 2007–2015 [2]:p.95; [41]:p.308. The government expects the healthcare services to increase the use of patient experiences as part of QI and states that patients are an unexploited resource in the QI. One motive behind the drive for enhanced patient involvement is improved outcome in which the incorporation of patient experiences is a means for improving quality:

*“High service quality is important for patients, users, and next of kin. We have to measure quality and make the results available for all. Users and next of kin often have good suggestions for improving the services. Systematic collection of user experiences is an important tool in quality improvement work and innovation”*[2]:p. 95 (translation made by authors).

Moreover, the NHCP (2011–2015) includes examples of successful service development projects where co-design involving patient interviews was used to map patients’ needs as a basis for improving service quality [2]:p.87. Patient experiences are also considered important in improving coordination of services and to increase transparency and improve information access for patients [2]:p.95, [41]:p.308. The NHCP (2011–2015) states the following:

“*There is need for improved knowledge about how and to what degree user involvement is accomplished, the effects of user involvement, and how the voice of the users can be made more explicit”*[2]:p.86 (translation made by authors)*.*

The National Strategy for Quality Improvement in Health and Social Services (2005–2015) [3] defines the Norwegian conceptualization of quality in healthcare:

“*based on meeting the demands of society, meeting legislative requirements, and providing users with the best possible services from a professional perspective. For health and social services, high quality means that the services: 1) Are effective, 2) Are safe and secure, 3) Involve users and give them influence, 4) Are coordinated and continuous, 5) Utilize resources efficiently, and 6) Are available and evenly distributed”*[3]:p.12.

The national strategy for QI emphasizes patient involvement and using patient experience. Several possible measures (direct, indirect, individual, and collective) to incorporate patient involvement and patient experiences in QI are listed as follows:

“…*develop effective methods for involving users in decision processes, improve users access to information about quality and safety, investigate measures to improve cooperation between users and providers, establish user oriented arenas for cooperation, further develop training and instruction programs for users in cooperation with user organizations, evaluate methods for improving user participation*) [3]:p.35.

Systematic collection of patient experiences and patient involvement at the individual and collective levels is stressed in the strategy; however, it places the responsibility of choosing the right measures and solutions for achieving the goal on the service providers [3].

### Context

In Table 2, we describe the context of the hospitals including location, size, structure, population, and number of staff.

**Table 2:** Description of hospital context

**Contextual aspect**	**Site A**	**Site B**
Localization	District, rural areas	Urban, city
Population served	107.000 inhabitants in the county; the city has 11.600.	490.000 inhabitants in the county; the city has 263.000.
Number of hospitals in the trust	3 (1 regional (Site A), 2 local).	Health trust owns 11 institutions, whereof Site B is the largest.
Teaching/non-teaching	Teaching hospital for nursing students	Teaching hospital for medical and nursing students, and other health care professions
Number of beds	300	1100 (related to acute services, not psychiatry)
Number of staff	2.336 employees, 1.988 full-time equivalents in the trust. Site A has 1.541 employees, hereof 179 physicians and 428 nurses	11.000 employees, 7.700 full-time equivalents. Site B has 4.259 nurses and 1.128 physicians
Organizational structure	Hierarchical	Flat

### Hospital practice – Site A

Site A is a relatively small rural hospital in the Norwegian context. For the past 6–7 years, Site A has been the subject of restructuring of its services, downsizing, and constant pressure to reduce costs. Quality champions among the top management started a structured approach to QI in 2005. The development of a new QI program in addition to a systematic use of available quality information were the initial steps of the improvement journey of Site A. Senior managers describe a systematic improvement journey based on projects driven by the need to control costs and to modernize the services according to national and regional expectations. QI is described from a holistic perspective in which budget management, patient safety, quality, effective use of resources, and high-quality employees are integrated aspects of managing the hospital. Management philosophy in the improvement strategy was to integrate top-down and bottom-up approaches. The multidisciplinary approach of the QI program and the involvement of all professions in problem identification and problem solving generated enthusiasm among the employees. Consequently, Site A had a strong QI leadership and a clear and well-organized structure. Indirect collective patient involvement is established by patients represented in an overall user panel at the hospital, in the overall quality committee, and in the steering committee of the QI program. Ideally, patient representatives are also expected to be included at each phase of the QI project. In addition, indirect patient involvement activities have been introduced at Site A, such as patient surveys (irregular) and a mailbox to collect patient experiences on the wards.

Despite the positive structural and leadership issues, our findings showed significant difficulties regarding incorporating collective patient involvement and patient experiences in QI in practice. As a senior manager said:

“I think it’s difficult to take advantage of the users’ involvement in QI. We try to integrate users in the steering group of the improvement program, users are represented in the quality committee and they can bring in their perspectives, but it is difficult in practice… It is difficult to involve users because the projects are so detailed, and the users often don’t have the qualifications to go into these details. They almost turn into a hostage left on the sideline, and they have no possibility of going into details and giving advice. We have 100.000 users, but the users involved in the projects are always the same people. We have no experience of users taking an active part and telling us what to do. They are pacified, and it is difficult”.

Another senior manager said:

“*I used to be represented in the user panel, but I asked for permission to leave, because I have too much to do. I think it took too much time, and we did all the talking. The users were only passive listeners*”.

A patient representative on the user panel described the same difficulties regarding the integration of patients in the improvement efforts at the hospital, but from a different perspective. The patient representative argued that the hospital did not take advantage of the user panel’s competence or provide the necessary training in QI of members to empower individuals and the panel as a whole. Despite a good collaborative climate between the hospital administration and the user panel, there appeared to be a lack of dialogue and interaction. According to the patient representative:

“If you want to achieve real user involvement, there is a need for a meeting point with the Board. The Board makes the decisions and provides the strategic direction…. It is about the Board. I can read what it says here: ‘the Board must make sure that experiences, needs, priorities, and views of patients and next of kin and their organizations are vital dimensions of planning and running the business’. Then I think: what kind of interaction are we having with the Board? We meet with the Board once a year, and it is really strange because we don’t have a real dialog with them, and I don’t feel they know anything about our competence and they don’t ask for our competence… and they don’t use our competence to improve service quality”.

The approach to patient involvement at Site A could be characterized as reactive, as service users were involved in QI, but their involvement was limited in accordance with a pre-defined agenda. There was a clear, formal acknowledgment of the need and expectations for using patient experience and patient involvement in QI on one hand, but also by difficulties and barriers to implementation on the other. Several senior managers expressed increased attention to patient involvement in QI and argued for a positive change in the organization to embed a stronger patient representative role. A senior manager said:

“*The user panel has changed from being a passive audience arguing for a smoker room or just passively listening to the CEO, to an audience engaged in developing and improving the organizing of the specialized healthcare services. Of course, their influences depend on the space we give them, the composition of the panel, and their qualifications*”.

At the same time, patient involvement and integration of patient experiences in QI was not being implemented consistently at the individual and collective level through the entire organization. Our findings indicate that patient involvement and use of patient experiences were not clearly apparent in clinical practice at the individual and the micro levels, although there was awareness of terms and clinical models stimulating patient involvement in clinical encounters. A medical doctor said:

*“Patient experiences are not sufficiently used in QI. One aspect is the direct feedback from the patient during consultations, meaning that before we start treatment we practice open conversations and adapt to patients’ needs and wishes. That’s how it should be, user involvement. But in fact it should be user co-decision and counseling from healthcare staff members.… I think patients should sign consents to diagnostic treatment and therapeutic methods. Norway is a developing country on user involvement*”.

A senior manager said:

“*Shared decision making, with its origin from the USA and England, is hot at the moment. We are entering a new era − the patients’ era – and this is one of the major challenges we are facing. I think the role of patients needs to change. Real user involvement is more than just telling the doctor to do what’s best for me as the patient. A lot of patients want to delegate decisions to the doctors”.*

Site A faced educational and cultural challenges relating to the integration of patient experiences in QI and individual involvement. Despite awareness of shared decision making and patient experiences in QI, they lack knowledge on how to collect and use patient experiences, and in combination with a lack of tools and physical and technological facilities to support leaders and staff, these conditions have hampered their integration to date. A senior manager said:

“I have a really bad conscience when I think of patient involvement in QI. We are really bad at it, and we know it. I really want to achieve real user involvement in practice not just on paper. But you know the regional health authority has developed an IT tool to use in user surveys, and the purpose is that we are going to apply it”.

The new IT tool was presented at a QI conference organized by Site A, including patient representatives and the regional health authority and will be implemented. The tool is supposed to enable the collection of patient experience data at the micro level (wards). An experienced physician at the maternity ward said:

“*We have conducted a few user surveys, but it was quite a while ago. But we are lucky that positive feedback is pouring in. People are sending post cards and letters, and they call and tell our nurses that they are really satisfied with the services we provided. From time to time you get the feeling that you only hear the negative feedback and it’s only feedback from the unsatisfied users. Anyway, it is a long time since we had a formal user survey. Back then the results were good, but I think it’s about time to conduct a new survey in a more objective way than the feedback from the users. Our services have changed a lot the past years – we have reorganized, patients have longer travelling distances, and we are actively using the patient hotel and day surgery. The feedback often concerns things that we are unable to do anything about, such as travelling distances or minor stuff such as more coffee in the waiting room. But we are not implementing any major changes based on user surveys or feedback from patients”.*

In the clinical micro systems of maternity and oncology at Site A, our findings show a culture of strong professional commitment to and enthusiasm for providing high-quality patient-centered care for the patients. Among the physicians, we found strong support for clinical effectiveness and having time for their patients as the basis of quality, while the nurses were more preoccupied with the patients’ needs. An example of the nurses’ focus was demonstrated as part of the restructuring process in the maternity micro system, during which the gynecology and maternity wards were merged. This resulted in the women having abortions located on the same corridor as the women giving birth. Several of the midwives and the gynecology nurses found the situation to be ethically challenging and disrespectful to the patients and their dignity. By engaging in the situation, being the patients’ advocates, and consulting the leaders, they were able to relocate the gynecology patients having abortions to another floor. By attempting to see the situation through the eyes of their patients, the nurses managed to spare these patients, who often face difficult situations, the experience of sharing a corridor filled with baby noises and happy families.

### Hospital practice – Site B

Site B is a large teaching hospital in a city in Norway. Site B is characterized by a flat, organizational structure focusing on delegation of responsibility, bottom-up leadership approaches, and a culture of empowerment. A senior manager said:

“*It is an important characteristic of our organization that ‘the power to define’ is delegated, in contrast to other hospitals that we can be compared to where the power to define is centralized in the organizations*”.

A department manager explained:

“*I think people need to feel ownership to produce quality. It is not just coming top-down*”.

QI is a management line responsibility, but the hospital has devoted resources to establish a section for patient safety, which is considered an important resource for Site B in promoting a systematic QI approach. There is an overall quality strategy, an overall quality committee, quality committees on the department level, a patient safety committee, and a user panel. The hospital seems to be undergoing a process in which patient involvement on a system (collective) level is increasing. Patients are represented in diverse projects at the hospital, and the patient representative explains that there has been a shift in interest regarding the patients’ voices in relation to being taken seriously as partners on the system level. The patient representative explained:

*“I think it is the participation in the projects that enables us to contribute to quality improvement. For example, we are represented in the project on reorganising the outpatient clinics in the main building. The project relates to patient safety as well as universal design and access and what is efficient patient treatment”*.

However, our findings suggest that patient involvement and patient experiences are not generally recognized as important aspects of QI processes. Whilst some informants explained the advantage of bringing the patients’ voices into projects, the active use of diverse methods, tools, and techniques for integrating patients and patient experiences in QI are lacking in practice. Even patient surveys are not occurring on a regular basis, and the results are viewed as too general (i.e. could not be broken down to specific clinical teams or wards). Senior managers pinpointed the need for external assistance and resources to conduct patient surveys. At Site B, senior managers are referring to the regional project in which a new IT tool for patient surveys is being developed by the regional health authority. Some interviewees also mentioned the value of listening to patient stories and giving patients time to tell their stories. Broadly, our findings nonetheless show that some of the informants view the patients’ perspective as too narrow, focusing too much on an individual’s own situation (i.e. they are not generalisable to other patients). A senior manager said:

“*It is important to integrate users in all types of projects. But it is difficult to find user representatives who are contributing to the project with constructive input. Our experience is that users are preoccupied with their own tragedy or their own patient history. They need the comprehensive perspective, and the user representatives with the capacity to apply a holistic perspective are really good. But our experience is that the user representatives often have a hidden agenda. I think we are asking for the professional user representative”.*

The role of the macro-level organizations’ contributions with tools and guidance and strong expectations for patient involvement in QI was regarded negatively. A senior manager said:

“I have talked to others about how to involve users, and it is difficult. The funniest thing is the Norwegian Knowledge Centre for Health Services and their philosophy regarding practice. They are separating evidence-based practice into three aspects, and user knowledge is one pillar in this philosophy. But except for user surveys, they are not promoting anything else to provide knowledge about the user aspect. How should we take advantage of the patient experiences and user involvement? They are just handing it over to the health services”.

Informants’ perception of quality at Site B is founded on evidence-based clinical effectiveness without any emphasis on patient experience in the improvement processes, neither directly/indirectly nor individually/collectively. The senior managers described how the hospital has built a culture focusing on the system perspective to QI, a systematic and evidence-based approach in order to engage all employees – especially doctors. There have constantly been quality champions among the clinicians at Site B. The influence and power of the clinicians, trust in evidence-based approaches, and conceptualization of quality in narrow terms as purely concerned with clinical effectiveness are highly evident in the micro system study in the maternity ward.

Site B has a strict and conservative policy regarding Caesarean-sections (C-sections) and promotes natural births, including in cases of twin births and breech position. Doctors and midwives argue in favor of natural birth, based on evidence of natural birth giving the best clinical effectiveness for the mother and the baby. Reference to patient involvement and patient experiences is not part of their C-section policy argumentation. Site B is the only hospital in the geographical area which restricts the patients’ ability to choosing another hospital if they prefer a less restrictive approach to C-sections. When exploring in more detail the patients’ experience of a strong tradition focusing on natural birth, we asked how employees deal with patients with birth anxiety who request an elective C-section. The doctors explain that they encourage good contact with the general practitioner (GP) of women with birth anxiety in order to offer these future patients the opportunity to come to the clinic for a meeting and conversations with the clinicians. A physician in specialization said:

“We try to involve these women in the process, at least the women who contact us. It is worse for the anxious women who don’t contact us; then we are unfamiliar with their fear. When our ‘machinery’ starts and we take them to the delivery rooms, we don’t change plans. We are not performing an acute C-section just because she doesn’t want to give birth. No, but it is not easy”

While Site B reports a low C-section rate and informants argue in favor of promoting natural birth as a mean for improving high quality in the maternity services, it traditionally reported high rates of severe perineal ruptures. Norway introduced the severe perineal rupture rate as a national quality indicator in 2011, and this topic receives increasing attention at the macro level. The Women’s Clinic at Site B started an improvement project to reduce the perineal rupture rate several years ago, due to instructions from the Board of Health in the county. Clinicians led the improvement project, and they implemented measures to improve diagnosing and changed procedures on the ward to always include two midwives during delivery. Important aspects in the delivery situation were the midwife’s support and competence, and specific training to reduce risk of perineal ruptures for the patients. The figures for perineal ruptures of midwife-led deliveries, regardless of delivery method, has declined from 3,5% to 2,4%.

## Discussion

The Norwegian political and regulatory environments of the outer context [28,42] have for several years applied strong expectations towards service providers at the meso and micro levels to involve patients in service improvement and link patient experience to QI. Macro-level expectations relate to all categories of patient involvement in Tritter’s conceptual framework [17]. Policy documents and regulation point to elements of individual, collective, direct, indirect, reactive, and to some degree proactive patient involvement. The emphasis on the future role of patients indicates intentions to develop patient involvement in a direction from reactive towards more proactive approaches in Norway as a response to more competent and well-informed patients and acknowledgment of the benefits from collaboration with patients and patients’ groups in service planning and development.

The Norwegian macro-level-enforced self-regulation approach to healthcare services [36,38] implies that the responsibility for developing tools and methods rests upon service providers, including the involvement of patients and incorporation of patient experiences as a vital resource in ongoing QI work. Our data suggest that this may be a challenging approach. Site B missed external support and resources to follow up on macro-level demands, and both hospitals embraced a new IT tool developed by the regional health authority, for collecting patient experiences. The hospitals in this study have systematically prioritized an indirect collective approach involving user panels as a means to involve patients in QI, and they have been less systematic in their use of patient experience surveys and providing opportunities for patients to give feedback on services received at the individual level. Tritter (2009) refers to user panels and surveys as involvement strategies that are indirect and operating at both the individual and collective level of services. The views of service users are sought at the two hospitals in order to inform clinical staff members and health care managers about the quality of service delivery, but decision making is still in the hands of health care staff and managers [17]. There are also examples of hospital staff members being concerned with services not attending to patients’ needs and preferences, but despite their concern, they are not involving patients in their QI initiatives. Seen from a multi-level perspective, there still remain unsolved questions as to how incentives, regulation, and policy influence real hospital practice in the relationship between service providers and patients as part of the QI. At the hospital (meso) level, we do not know which national policy levers (policy expectations, statutory law/regulation, sanctions, guidance, publication of information, etc.) work best to improve the hospitals’ patient involvement and use of patient experience in QI, meaning that this is a relatively evidence-light zone in which to make policy decisions [24,42].

Despite some differences between the two hospitals in this study, patient involvement and patient experiences in QI work were underdeveloped at both sites, meaning that they do not meet governmental expectations. We believe there are multiple causes and explanations for the lack of patient involvement and patient experience focus in day-to-day hospital practices in Norway. The most evident interpretations are related to the interactions between various processes within the inner context of the hospitals [28]; the politics, leaders’ priority, and attention to patient experiences and patient involvement [43,44]; the resource situation (budget and personnel); the level of knowledge and competence regarding patient experience and patient involvement as part of QI [28,44,45]; and cultural and power issues [7,46]. The inner context of the organizations differs significantly: Site A is a small hospital with a hierarchical structure that adopts a relatively top-down leadership approach combined with management enthusiasm for QI. Site A has approached QI in a systematic manner at the collective level, as patient involvement is integrated as part of the formal QI structure. Despite this, the patient participants have a limited influence on decision making due to lack of dialogue and interaction with the hospital administration. Using the conceptual frame form Tritter [17], this method of involving service users is reactive, meaning that patients are involved but only as a response to specific and predefined agendas which they cannot influence. Competence, tools, and methods are lacking in order to integrate patient experiences and involve patients in direct service improvement at an individual and organizational level. Site B is a large hospital with a flat structure that relies strongly on clinical empowerment and bottom-up leadership approaches. At Site B, patient involvement and patient experiences are integrated as part of the formal QI structure and in user panels, but this input is more or less absent in the QI efforts of clinical practice. The strong professional culture and organizational identity at Site B conceptualizes quality largely in terms of clinical effectiveness, which could be counterproductive in terms of recognizing the importance of the patient perspective and promoting shared decision making in clinical encounters (such as decision making about C-sections) and QI work. The conservative C-section policy and the interpretation of quality largely as clinical effectiveness also illustrates the potential inherent trade-offs in balancing clinical effectiveness, patient safety, and patient experiences when improving quality. There are instances in which all three quality dimensions can be improved, but in the case of Site B, the data show that patient experience can be traded off in favor of clinical effectiveness when professionals define quality as natural birth for patients.

At both sites, current measures of patient experience (patient surveys) are not used meaningfully or systematically at the meso or micro level for a range of reasons, but significantly because they are not yet seen as clinically relevant at a service level and are captured too infrequently [10,12]. The routine use of surveys is also not supported by the inner or outer context, and they are time consuming. There are examples of how patient stories affect the meso-level managers, and all clinical personnel express a preoccupation with providing high-quality care and serving patients’ needs. However, patient voices and their service experiences are not seen as a valid source of input for QI work. At both sites, interviewees argued in favor of the “professional patient representative” as being the key to bringing in the patient perspective and additional opinions; i.e. patients who are “educated” to participate in QI projects (despite the growing body of literature suggesting several significant limitations to this approach [47]). Other methods for capturing patient experiences, such as stories and narratives, are not considered as beneficial to QI in the long run. There also seems to be a lack of resources, competence, and priority at the microsystem level, shaped to a large extent by the struggle of handling daily operations, continuous organizational changes, and budget cuts.

Our findings of an implementation gap between macro-level expectations and day-to-day practice at the meso and micro levels echo those of other studies [48,49]. Although national policymakers may advocate for certain policies and develop and support them centrally (and perhaps disseminate them vertically for adoption at the local level), and even if there is strong alignment in the values underpinning both central and local policymaking on, as in this example, patient involvement and patient experience, this does not guarantee local enactment of those policies.

## Conclusions

This study has shown how the Norwegian macro-level expectations, highlighting the importance of the patients’ role in service planning and improvement, contrast starkly with day-to-day hospital practice. Macro-level policy and regulation expect a wide range of patient involvement at the individual and collective levels, involving both direct and indirect approaches. More emphasis is also given to the new role of patients in a proactive manner, in which patients are conceptualized as having a more important role in the current National Healthcare Plan period (2011–2015). Meso-level practice at both sites prioritize indirect collective patient involvement in terms of the user panels, while the micro level gives priority to indirect individual patient involvement by infrequent patient surveys [17].

It is worth noting how this cross-level exploration [50] of patient experiences has revealed a gap between macro-level goals and expectations and what is happening in real-world hospital practices. The macro-level goals can be interpreted as aspirational and lacking in instrumentality, but this deficiency is according to the current Norwegian model of enforced self-regulation [36-38]. Based on this study, there is a need for acknowledgment at the macro level that aspirational goals seem difficult to operationalize and implement in the current regulatory regime, and there is a need for more knowledge on the role of the macro-level institutions in assisting in the implementation of government expectations for QI in health care.

At the current stage of the QI journey of these site hospitals, we would argue for increased priority given to patient involvement and patient experiences at the meso level in order to focus attention among board members and managers with the authority to put the topic on the organizations agendas and allocate sufficient resources to this effort. In addition, there is a need for quality champions in each of the hospitals to integrate patient experience within the prevailing conceptualizations of quality, alongside clinical effectiveness and patient safety. The relative lack of expertise in Norway in adapting and implementing tools and methods for improving patient involvement and patient experiences at the meso and micro levels marks a call for, firstly, health care macro-level policymakers and hospital leaders to look to the experiences of other industries and countries that have successfully integrated user experiences into ongoing QI work, and secondly, for hospital managers to design and implement cultural, emotional, and educational strategies to help their staff members recognize and value the important contribution that patient involvement and patient experiences can make to improve healthcare quality efforts. There is a need for the development of new knowledge and a culture to support patient involvement and a use of patient experiences, involving a more comprehensive repertoire of involvement strategies in QI work as one of multiple goals within hospital practices.

## Endnote

^a^Mental Health Act [51] regulates obligations to include patient involvement in mental health.

## Competing interests

The authors declare that they have no competing interests.

## Authors’ contributions

SW contributed in terms of the study design, acquisition of data, analysis and interpretation of data, manuscript draft, and revision. KAA contributed to the study design, acquisition of data, analysis and interpretation of data, and manuscript revision. MSt contributed to the interpretation of data, manuscript draft, and revision. MTG contributed to the acquisition of data, analysis and interpretation of data, and manuscript revision. MSo and SH contributed to the coordination of data collection and manuscript revision. GR contributed to the conception and design of the study, manuscript draft, and revision. NF contributed to the conception and design of the study and manuscript revision. All authors have approved the final manuscript.

## Pre-publication history

The pre-publication history for this paper can be accessed here:

http://www.biomedcentral.com/1472-6963/13/206/prepub

## References

[B1] Report no. #47 (2008–2009). Report to Parliament: Coordination reform; Right treatment − the right place − at the right time http://www.regjeringen.no/nb/dep/hod/dok/regpubl/stmeld/2008-2009/stmeld-nr-47-2008-2009-.html?id=567201

[B2] Report no.#16 (2010–2011): Report to Parliament: National Health Plan (2011–2015) http://www.regjeringen.no/nb/dep/hod/dok/regpubl/stmeld/2010-2011/meld-st-16-20102011.html?id=639794

[B3] Directorate of Health National strategy for quality improvement in health and social services (2005–2015) 2005 http://www.helsedirektoratet.no/english/publications/and-its-going-to-get-better/Sider/default.aspx

[B4] IOM (Institute of Medicine) Crossing the quality chasm; a new health system for the twenty-first cenury 2001 Washinghon. DC: National Academy Press

[B5] Bate P Robert G Bringing user experience to healthcare improvement 2007 New York: Radcliffe Publishing

[B6] Vincent C Patient safety 2010 Oxford: Wiley- Blackwell

[B7] Ocloo JE Fulop NJ Developing a ‘critical approach’ to patient and public involvement in patient safety in the NHS: Learning lessons from other parts of the public sector? Health Expectations 2011 10.1111/j.1369-7625.2011.00695.x PMC5060634 21711471

[B8] Madden PB Davies EA Reporting cancer patients’ experiences of care quality improvement: analysis of 2000 and 2004 survey results for South East England Journal of Evaluation in Clinical Practice 2010 16 4 776 783 10.1111/j.1365-2753.2009.01192.x 20545797

[B9] Davies E Shaller D Edgman-Levitan S Safran D Oftedahl G Sakowski J Cleary PD Evaluating the use of a modified CAHPS® survey to support improvements in patient-centred care: Lessons from a quality improvement collaborative Health Expectations 2008 11 2 160 176 10.1111/j.1369-7625.2007.00483.x 18494960 PMC5060434

[B10] Coulter A Fitzpatric R Cornwell J The point of care: measures of patients’ experience in hospital: purpose, methods and uses 2009 London: The Kings Fund http://www.kingsfund.org.uk/sites/files/kf/Point-of-Care-Measures-of-patients-experience-in-hospital-Kings-Fund-July-2009_0.pdf

[B11] Lombarts MJ Rupp I Vallejo P Sunol R Klazinga NS Application of quality improvement strategies in 389 European hospitals: results of the MARQuIS project Quality and Safety in Health Care 2009 18 suppl 1 i28 i37 10.1136/qshc.2008.029363 19188458 PMC3269892

[B12] Davies E Cleary PD Hearing the patient’s voice? Factors affecting the use of patient survey data in quality improvement Quality & Safety in Health Care 2005 14 428 432 10.1136/qshc.2004.012955 16326789 PMC1744097

[B13] Bate P Robert G Experience based design: from redesigning the system around the patient to co-designing services with the patient Quality and Safety in Health Care 2006 15 5 307 310 10.1136/qshc.2005.016527 17074863 PMC2565809

[B14] Légaré F Ratté S Stacey D Kryworuchko J Gravel K Graham ID Turcotte S Interventions for improving the adaption of shared-decision making by healthcare professionals (Review) The Cochrane Database Of Systematic Reviews 2010 http://onlinelibrary.wiley.com/doi/10.1002/14651858.CD006732.pub2/abstract 10.1002/14651858.CD006732.pub2 20464744

[B15] Lewin S Skea Z Entwistle VA Zwarenstein M Dick J Intervention for providers to promote a patient-centered approach in clinical consultations (Review) The Cochrane Database Of Systematic Reviews 2001 4 CD003267 http://onlinelibrary.wiley.com/doi/10.1002/14651858.CD003267/full 10.1002/14651858.CD003267 11687181

[B16] Berwick DM What ‘patient Centered’ should mean: confession of an extremist Health Affairs 2009 28 4 555 565 10.1377/hlthaff.28.4.w555 19454528

[B17] Tritter JQ Revolution or evolution: the challenges of conceptualizing patient and public involvement in a consumerist world Health Expectations 2009 12 275 287 10.1111/j.1369-7625.2009.00564.x 19754691 PMC5060496

[B18] Storm M Edwards A Models of user involvement in the mental health context: intentions and implementation challenges Psychiatr Q 2012 10.1007/s11126-012-9247-x 23076765

[B19] Thompson AGH The meaning of patient involvement and participation in health care consultations: A taxonomy Social Science & Medicine 2007 64 6 1297 1310 10.1016/j.socscimed.2006.11.002 17174016

[B20] Crawford MJ Rutter D Manley C Weaver T Bhui K Fulop N Tyrer P Systematic review of involving patients in the planning and development of health care BMJ 2002 325 7375 1263 1265 10.1136/bmj.325.7375.1263 12458240 PMC136920

[B21] Robert G Ziebland S, Calabrase J, Coulter A, Locock L Participatory action research: Using experience-based co-design (EBCD) to improve health care services Understanding and using experiences of health and illness Oxford: Oxford University Press in press

[B22] Foot C Cornwell J Improving patients’ experiences: an analysis of the evidence to inform future policy development 2010 London: The King’s Fund

[B23] Piper D Iedema R Merrick E Perrott B Emergency Department Co-Design Program 1 Stage 2 Evaluation Report - Final Report to 2010 Health Services Performance Improvement Branch, NSW Health, Sydney: Centre for Health Communication, University of Technology

[B24] Robert G Cornwell J ‘What matters to patients’? Policy Recommendations. A report for the Department of Health and NHS Institute for 2012 King’s College London and the King’s Fund: Innovation & Improvement, London

[B25] Robert GB Anderson JE Burnett SJ Aase K Andersson-Gare B Bal R Calltorp J Nunes F Weggelaar AM Vincent CA Fulop NJ QUASER team A longitudinal, multi-level comparative study of quality and safety in European hospitals: the QUASER study protocol BMC Health Services Research 2011 11 285 10.1186/1472-6963-11-285 22029712 PMC3212959

[B26] Burnett S Renz A Wiig S Fernandes A Weggelar AM Calltorp J Anderson JE Robert G Fulop N Prospects for comparing European hospitals in terms of quality and safety: lessons from a comparative study in five countries International Journal for Quality in Health Care10.1093/intqhc/mzs079 PMC3557961 23292003

[B27] House R Rousseau DM Thomas-Hunt M The meso-paradigm: a framework for the integration of micro and macro organization Research in organizational Behaviour 1995 17 71 114

[B28] Bate SP Mendel P Robert G Organizing for Quality, The Improvement journeys of leading hospitals in Europe and United States 2008 Oxford: Radcliffe Publishing

[B29] Lincoln YS Guba EG Naturalistic Inquiry 1985 Beverly Hills, California: Sage

[B30] Seale C The quality of qualitative research 1999 London: Sage

[B31] Krein SL Damschroder LJ Kowalski CP Forman J Hofer TP Saint S The influence of organizational context on quality improvement and patient safety efforts in infection control: a multi-center study Social Science & Medicine 2011 71 9 1692 1701 20850918 10.1016/j.socscimed.2010.07.041

[B32] Patient Right Act, 1999 http://lovdata.no/all/nl-19990702-063.html

[B33] Specialist Health Care Act, 1999 http://www.lovdata.no/all/nl-19990702-061.html

[B34] Health Trust Act, 2001 http://www.lovdata.no/cgi-wift/wiftldles?doc=/app/gratis/www/docroot/all/nl-20010615-093.html&emne=HELSEFORETAKSLOV*&

[B35] Health and Care Service Act, 2011 http://www.lovdata.no/all/hl-20110624-030.html

[B36] Wiig S Contributions to risk management in the public sector PhD thesis 2008 Stavanger: University of Stavanger, Faculty of Social Sciences

[B37] Wiig S Quarts J Plessen CV Harthug S Soares G Bérenguer G Organizing for quality and safety in health care – The Norwegian case Advances in safety, reliability and risk management 2011 London: Taylor & Francis Group

[B38] Braut GS Molven O, Ferkis J Legal requirements related to governance of health services Healthcare, Welfare and Law, Health legislation as a mirror of the Norwegian welfare state 2011 Oslo: Gyldendal Akademisk 129 138

[B39] Supervisory Act, 1984 http://www.lovdata.no/all/hl-19840330-015.html

[B40] Internal Control for Health Services, 2002 http://www.lovdata.no/for/sf/ho/xo-20021220-1731.html

[B41] National Health Plan (2007–2010) http://www.regjeringen.no/upload/HOD/Sykehus/Nasjonal_helseplan_Sartrykk.pdf

[B42] Stevens DP Shojania KG Tell me more about the context, and more BMJ Quality & Safety 2011 20 7 557 559 10.1136/bmjqs-2011-000206 21685496

[B43] Künzle B Kolbe M Grote G Ensuring patient safety through effective leadership behaviour: A literature reivew Safety Science 2010 48 1 17 10.1016/j.ssci.2009.06.004

[B44] Storm M Service user involvement in in-patient mental health services PhD Thesis 2011 Stavanger: University of Stavanger, Faculty of Social Sciences

[B45] Storm M Hausken K Knudsen K Inpatient service providers’ perspectives on service user involvement in Norwegian Community Mental Health Centers International Journal of Social Psychiatry 2011 57 6 551 563 10.1177/0020764010371270 20610463

[B46] Ocloo JE Harmed patients gaining voice: challenging dominant perspectives in the construction of medical harm and patient safety reforms Social Science & Medicine 2010 71 3 510 516 10.1016/j.socscimed.2010.03.050 20554367

[B47] Van de Bovenkamp HM Trappenburg MJ Reconsidering patient participation in guideline development Health Care Analysis 2009 17 98 216 10.1007/s10728-008-0099-3 PMC2725277 19101804

[B48] Exworthy M Berney L Powell M How great expectations in Westminster may be dashed locally: The local implementation of national policy on health inequalities Policy & Politics 2002 30 1 79 96 10.1332/030557302250158423745170

[B49] Bate SP Robert G Gabbay J Gallivan S Jit M Le May A Pope C Utley M The development and implementation of NHS Treatment Centres as an organisational innovation Report for the National Co-ordinating Centre for NHS Service Delivery and Organisation R&D (NCCSDO) 2007 London: HMSO

[B50] Hackman RP Learning more by crossing levels: evidence from airplanes, hospitals, and orchestras Journal of Organizational Behaviour 2003 24 905 922 10.1002/job.226

[B51] Mental Health Act 1999 http://www.lovdata.no/cgi-wift/wiftldles?doc=/app/gratis/www/docroot/all/nl-19990702-062.html&emne=PHLSVL%5D

